# Health4Life: a digital intervention to promote healthy diet and movement behaviours from the start of life: protocol for a hybrid effectiveness-implementation study

**DOI:** 10.1186/s12889-026-28866-0

**Published:** 2026-08-03

**Authors:** Christina Alexandrou, Marie Golsäter, Marie Löf, Kylie D Hesketh, Vicki Brown, Anna Strid, Jennifer Gransten, Henrike Häbel, Christine Delisle Nyström

**Affiliations:** 1 Department of Medicine Huddinge, Karolinska Institutet, Neo, Huddinge, 141 83 Sweden; 2 Department of Nursing, CHILD Research Group, School of Health and Welfare, Jönköping University, Jönköping, 551 11 Sweden; 3 The Child Health Services and Futurum – Academy for Health and Care, Region Jönköping County, Jönköping, 551 85 Sweden; 4 Deakin Institute for Physical Activity and Nutrition, Deakin University, Geelong, 3220 Australia; 5 Institute for Health Transformation (IHT), Deakin Health Economics, Deakin University, Geelong, 3220 Australia

**Keywords:** Child health services, Diet, Digital health, Infancy, mHealth, Physical activity, Randomized controlled trial, Screen time, Sleep

## Abstract

**Background:**

Globally and in Sweden childhood overweight and obesity remain major public health issues. The first 1000 days (i.e., conception-24 months) has been identified as the period of greatest developmental plasticity, with the maximum capacity to influence health throughout life. As obesity-related behaviours, including excessive screen time and poor dietary habits, have been found to establish in infancy and continue into early childhood, this demonstrates the need for early life health promotion interventions. Therefore, the aim of this study is to evaluate the effectiveness of and explore the implementation of a digital intervention (Health4Life app) designed to support parents in creating healthy diet and movement behaviours from the start of their infant’s life, to promote health and prevent overweight and obesity.

**Methods:**

A two-arm parallel open-label randomized controlled trial, using a hybrid type I implementation-effectiveness design will be conducted. Families will be recruited at the 6-week routine visit at participating child health service (CHS) centers (*n* = 504). If parents’ consent to participate families will be randomized in a 1:1 ratio into the control or intervention group. Both groups receive standard care; however, the intervention group also receives access to the 16-month Health4Life app-based intervention. Primary outcomes are children’s dietary intake (questionnaire), physical activity and sleep (accelerometry), and screen time (questionnaire). Secondary outcomes are children’s body mass index z-scores (measured at CHS) and parental self-efficacy for promoting health behaviours (questionnaire). The implementation outcomes being explored are acceptability, appropriateness, and feasibility according to CHS nurses (semi-structured interviews) as well as parental satisfaction and usage of the Health4Life app (questionnaires and semi-structured interviews).

**Discussion:**

An important mission of CHS is to support parents in establishing healthy diet and movement behaviours, and the Health4Life app is designed to support this by providing early and tailored guidance to families. If shown to be effective as well as acceptable, appropriate and feasible, Health4Life has the potential to be implemented as a complement to CHS nurses’ routine work to promote children’s health and development in accordance with the Swedish national child health monitoring program.

**Trial registration:**

This trial was registered at Clinicaltrials.gov (ID NCT06798597) on January 29th, 2025.

**Supplementary Information:**

The online version contains supplementary material available at https://doi.org/10.1186/s12889-026-28866-0.

## Background

Childhood overweight and obesity remain major public health issues globally. In 2024, ~ 35 million children under the age of five were overweight [1]. Sweden is no exception, with ~ 8% of 1.5-year-olds and 12% of four-year-olds being classified as having overweight or obesity [2]. Although some recent evidence suggests that the prevalence of overweight and obesity in 4-year-olds in Northern Sweden is decreasing [3], the prevalence remains much too high. According to the Swedish National Dietary Survey from 2024 only 8% of four-year-olds met the recommendation of 400 g of fruit and vegetables each day and over 50% consumed more added sugar than is recommended [4]. With regards to 1.5-year-olds, over 10% of parents reported that their child consumed candy, snacks, ice cream or soft drinks and over 25% reported that their child ate sweet bakery products at least once per week [4]. Additionally, the 2024 Generation Pep report, which included 7067 Swedish children and adolescents aged 4–17 years, found that 14% of parents of children aged four to six years reported that their child had spent three hours or more on a screen the previous day [5]. As higher levels of screen time have been found to be associated with greater obesity and adiposity, a higher energy intake, and a less healthy diet quality in children [6], there is a need to target these behaviours early. Furthermore, given that the majority of weight gain in adolescents with overweight and obesity has been found to occur between the ages of two and six years [7], this further highlights the importance of early life interventions aimed at establishing healthy diet and movement behaviours from the start of life.

The first 1000 days (i.e., from conception to 24 months of age) has been found to be the period of greatest developmental plasticity and with the maximum capacity to influence health throughout life [8]. Obesity-related behaviours including excessive screen time and poor dietary habits are established in infancy and continue into early childhood [9, 10]. To date, in Sweden, only one study has evaluated an obesity prevention intervention starting in infancy (at 9–10 months), where the intervention was based on child health service (CHS) nurses applying motivational interviewing to promote healthy dietary and physical activity behaviours. Post-intervention only weak effects regarding dietary behaviours were observed, which the authors attribute to imperfect intervention implementation [11]. However, in other countries early intervention has proven successful [12].

Digital health tools and services have become important avenues to provide information and support to address health needs in a variety of populations. To date, digital interventions have been shown to be effective in promoting physical activity and healthy eating as well as reducing sedentary behaviour in children and adults [13]. Additionally, a systematic review found that digital nutrition interventions targeting parents have been successful in improving nutrition outcomes in children [14]. In Sweden, the Mobile-based Intervention Intended to Stop Obesity in Preschoolers (MINISTOP), is a promising 6-month mobile health (mHealth) obesity prevention program with the overall aim to support parents to improve diet and physical activity behaviours in preschool aged children (2.5 to 4 years) [15, 16]. In the MINISTOP 2.0 trial where MINISTOP was delivered by CHS nurses, children in the intervention group compared to the control group reported statistically significant lower intakes of sweet and savoury treats, sweet drinks, and screen time post-intervention. Furthermore, parents in the intervention group reported statistically significant higher scores for parental self-efficacy for promoting both healthy diet and physical activity behaviours in their child [16]. Additionally, to build on this concept, we have conducted qualitative interviews with Swedish parents with an infant aged 2–12 months and CHS nurses to investigate the potential of a mHealth intervention to support parents in creating healthy diet and movement behaviours from the start of life. Swedish parents were eager for continuous support and wished to be able to find answers to their questions immediately when they arose. Parents also expressed that an evidence-based app could enhance the care they are currently receiving from CHS [17]. CHS nurses expressed that digitalization is the way of the future and were positive to the idea of introducing an app promoting healthy diet and movement behaviours. The nurses viewed the potential app as an avenue to convey evidence-based information to parents between individual visits [18].

Currently, in Sweden, early infancy represents an underexplored period for parent-focused interventions aimed at promoting health diet and movement behaviours. As 99% of all children in Sweden attend CHS [19], this provides an ideal setting for the delivery of a mHealth intervention to support parents in their efforts to establish healthy diet and movement behaviours from the start of their infant’s life.

## Aims

This study protocol describes the methodology and design of the Health4Life randomized controlled trial utilizing a hybrid type I effectiveness-implementation study design. The overall aim is to evaluate whether a digital tool (the Health4Life app), can be used to promote healthy diet and movement behaviours starting in early infancy. More specifically, we aim to:

(i) evaluate the effectiveness of the Health4Life app on: (a) children’s key dietary indicators, i.e., fruit and vegetables, sweet and savoury treats, and sweet drink intake (primary outcome); (b) children’s physical activity, i.e., time spent in total physical activity (primary outcome); (c) children’s screen time (primary outcome); (d) children’s sleep (primary outcome); (e) children’s body mass index (BMI) z-scores (secondary outcome); (f) parental self-efficacy for promoting healthy diet and physical activity behaviours (secondary outcome).
(ii) evaluate the cost-effectiveness of the Health4Life app.
(iii) explore the implementation of the Health4Life app regarding perceived acceptability, appropriateness, and feasibility among CHS nurses and parents.

## Methods

### Study design

This study utilizes a hybrid type 1 effectiveness-implementation design [20]. A mixed-methods approach will be used to collect data and the `Outcomes for Implementation Research´ evaluation framework will be used [21]. A two-arm parallel open-label randomized controlled trial will investigate the effectiveness of the Health4Life app on children’s health behaviours, BMI z-scores as well as parental self-efficacy for promoting healthy diet and physical activity behaviours. Furthermore, implementation will be explored with regard to parents’ and CHS nurses’ perceived acceptability, appropriateness, and feasibility of the Health4Life app. This protocol adheres to the SPIRIT 2013 [22] statement and the CONSORT – EHEALTH checklist (v.1.6.1) will be used to describe the intervention [23].

### Intervention – the Health4Life app

**Fig. 1:**
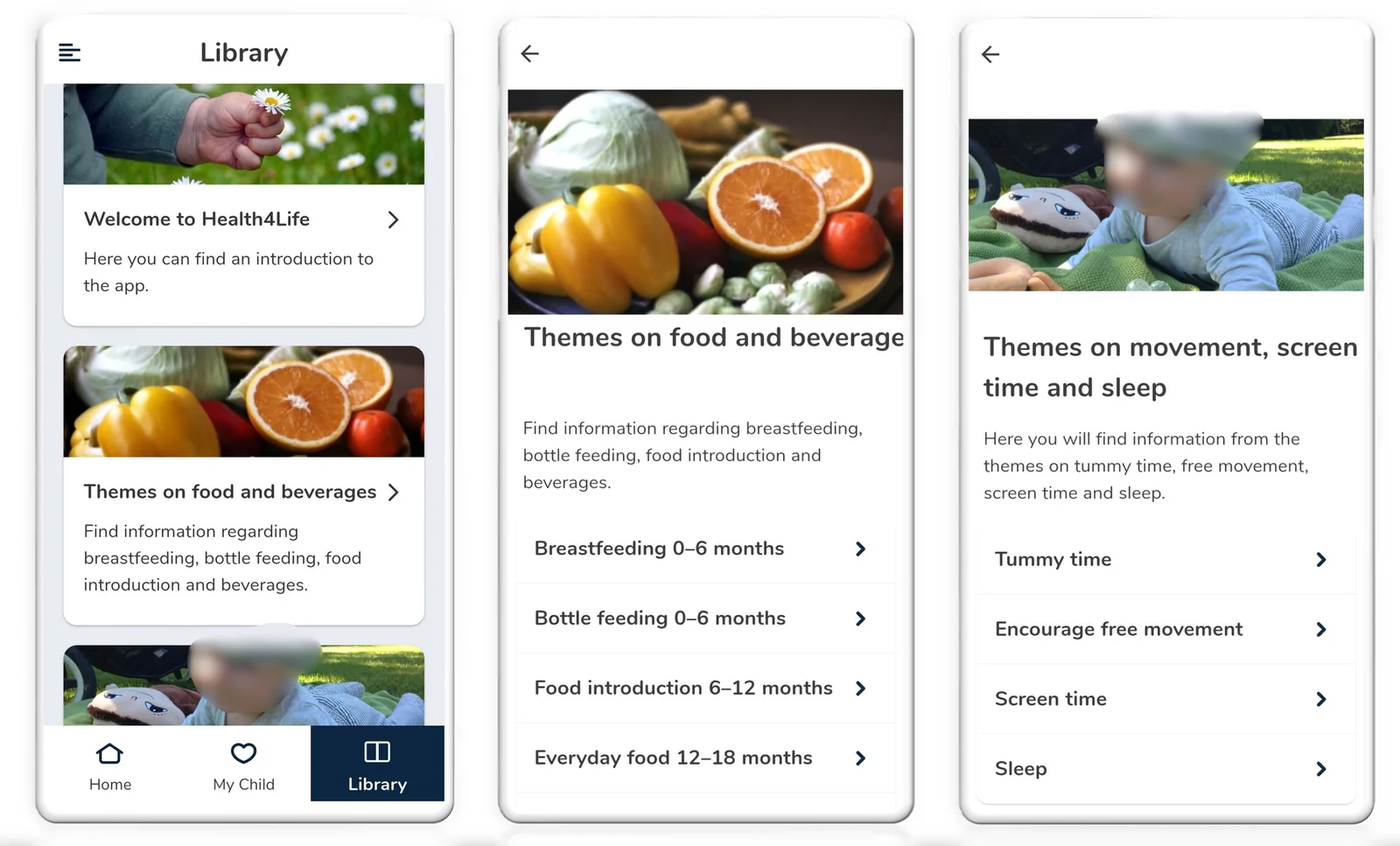
Screenshots from the Health4Life app showing the welcome screen as well as the library

The Health4Life app is a 16-month parent-focused comprehensive digital intervention supporting parents to establish healthy diet and movement behaviours early in life to promote health and prevent the development of overweight and obesity. Figure 1 shows screen shots from the Health4Life app. The Health4Life app builds on the anticipatory guidance approach [24], which is recognized as an important component of pediatric practice. By utilizing this approach, the Health4Life app will work proactively to support parents through preparing them about what to expect and how to manage forthcoming situations.

#### Development of the intervention

The Health4Life app uses the technical platform delivered by Cuviva AB (Stockholm, Sweden) and is available for both iOS and Android. The Health4Life intervention’s format and content is partially based off the INFANT program from Australia which is also delivered through their CHS equivalent utilizing the anticipatory guidance approach [25, 26]. The development team for the Health4Life intervention consisted of professionals with vast experience in nutrition, breastfeeding, physical activity, psychology, pediatrics, screen time, dentistry, and speech therapy. A co-design process following the Double Diamond Framework [27] was used to develop and adapt the content for the Health4Life app. This process consisted of repeated focus-groups with CHS nurses and a pediatric dietician (*n* = 5) working in CHSs as well as repeated semi-structured interviews with parents of young children aged 0 to 24 months (*n* = 5) (unpublished data). The development of the Health4Life intervention content was done in an iterative manner, where we received feedback from both healthcare professionals and parents of young children throughout the development process. In a second step 11 parents and eight healthcare professionals tested the app for a two-week period and provided feedback on the content and layout. Thereafter, final modifications were made to the Health4Life app based on the feedback obtained.

#### Content and use of the intervention

The Health4Life app consists of a comprehensive program with evidence-based information related to creating and promoting healthy eating, movement, and screen time behaviours for young children from two to 18 months of age [28–33]. The information content in the app consists of four components: general information (library), home screen, push notifications, and quizzes with subsequent feedback. The app comprises of ten themes: breastfeeding, bottle feeding, food introduction 6–12 months, everyday food 12–18 months, start your fruit and vegetable journey, drinks 0–18 months, tummy time, encourage free movement, screen time, and sleep. Furthermore, within the library there are additional themes on child development (divided into two-month intervals from two to 18 months of age), dental care, growth curve, and how to use sign language as a support for communication. In addition, there is a collection of ideas for activities promoting active play divided into two-month intervals from two to 18 months of age. There is also a recipe bank containing healthy family-friendly meal and snack ideas. Within the recipe bank there is information on how to make your own baby food and how to use up leftovers. Finally, there are numerous videos by a child dietician and a speech therapist answering commonly asked questions that parents have regarding child feeding, oral motor development as well as food textures.

The home screen of the Health4Life app changes every week and highlights a new theme, a component within a theme or an active play idea/recipe relevant to the age of the child. Furthermore, once per week parents will receive a push notification with a short message encouraging them to go into the app. These push notifications consist of reminders for tummy time, active play ideas, recipes, or basic facts in line with the age of the child.

Finally, the feedback component comprises of six quizzes allowing parents to receive knowledge through feedback on their current practices. The titles of the six quizzes are: (i) Is your baby ready for other foods?, (ii) Food introduction – what do you serve?, (iii) Healthy eating habits, (iv) What do your mealtimes look like?, (v) Play and free movement for babies who cannot sit up, and (vi) Play and free movement for babies who can sit up. Each quiz contains between three to nine multiple choice questions, where there are no right or wrong answers. Feedback is provided for every question and is based on the parent’s answer. The quizzes can be taken as many times as a participant likes.

### Intervention effectiveness evaluation

#### Participants and recruitment

**Fig. 2:**
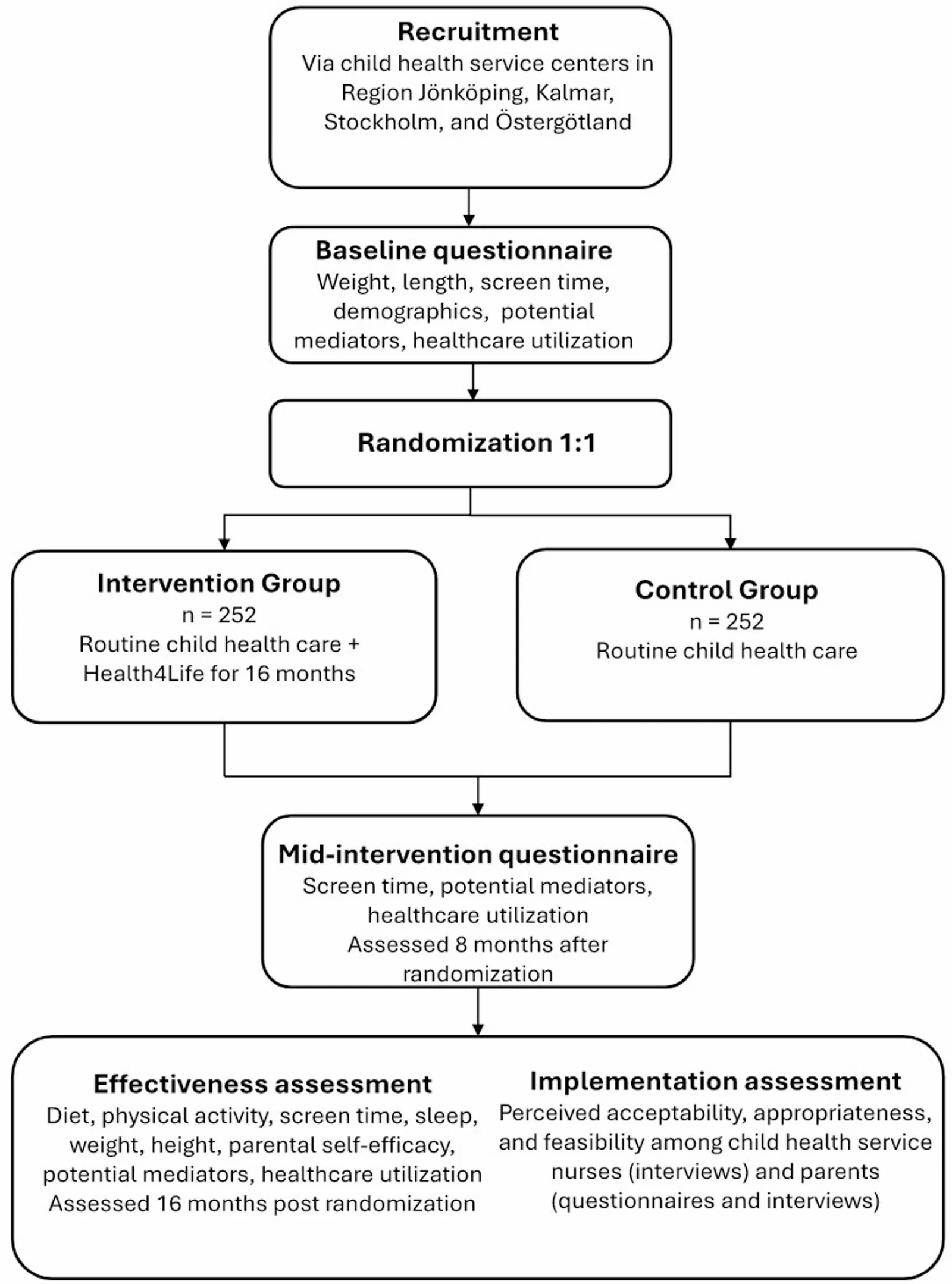
Flowchart of the Health4Life trial

**Fig. 3:**
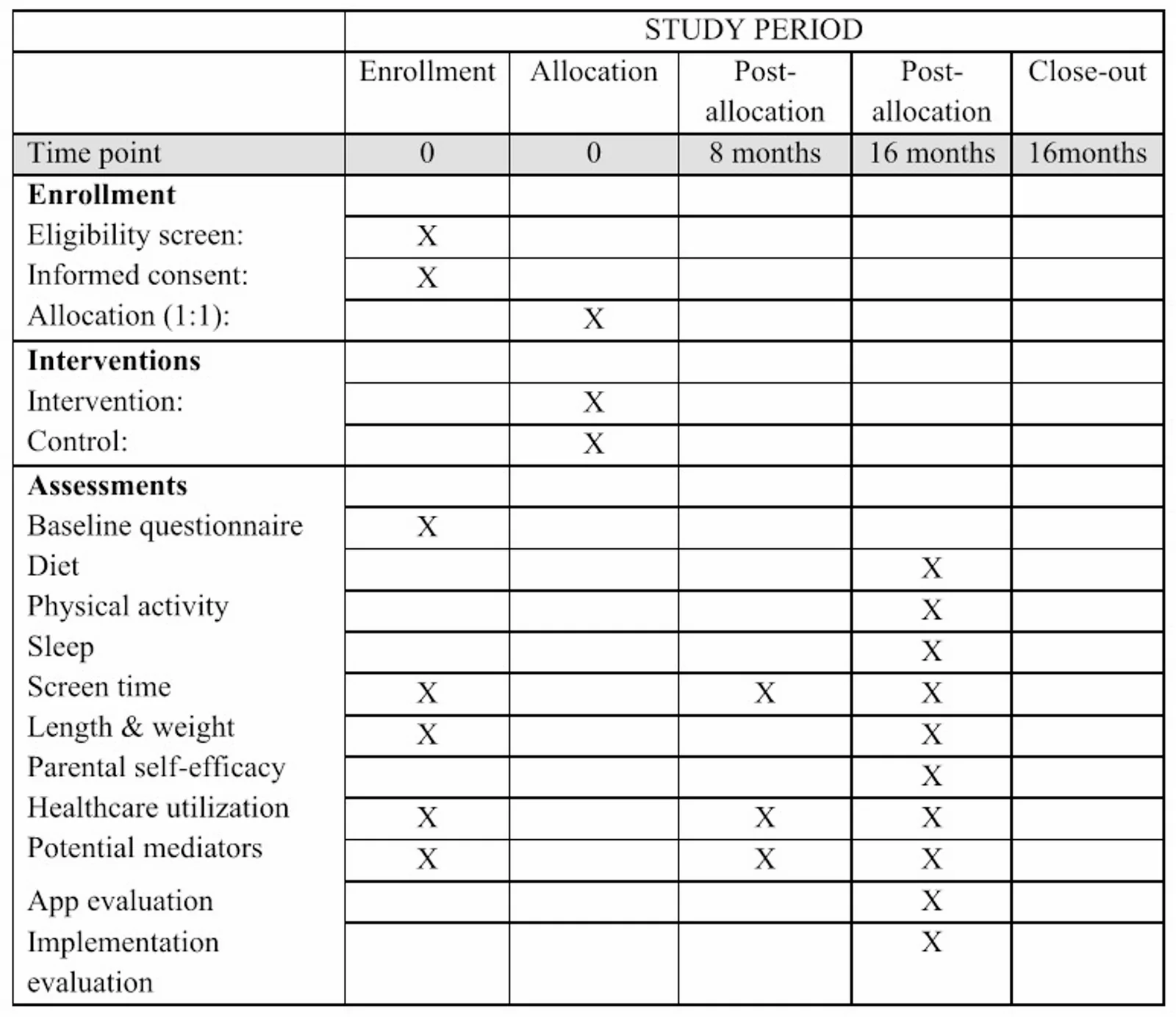
Participant timeline according to SPIRIT

All families who attend the routine 6-week visit at participating CHS centers in Regions Jönköping, Kalmar, Stockholm, and Östergötland will be asked if they are interested in participating in the Health4Life trial by their CHS nurse. If they are interested, they will scan a QR-code and provide their contact information to the research team (name, phone number, and email address) and the full study information will automatically be sent via email to them. Thereafter, a member of the research team will contact the interested parents to explain the study verbally and answer any questions they may have. If the parents want to participate, digital informed consent will be sent via SMS or email, and both parents will sign using their BankID (a secure digital signature). After receiving informed consent from both parents, the digital baseline questionnaire will be sent to the participating parent’s email. The parents decide themselves who will fill in the questionnaire and the same parent will fill in the questionnaire at all timepoints. To be included in this study parents need to have an infant between five and eleven weeks and one of the parents needs to be able to speak Swedish or English sufficiently well to provide informed consent and to make full use of the Health4Life app. There are no other exclusion criteria. Figure 2 presents the flowchart for the Health4Life trial and Fig. 3 shows the participant timeline.

#### Randomization

After completing baseline measurements, families will be randomly allocated to the control group (routine care from CHS) or the intervention group (routine care from CHS + access to the Health4Life app). Participants will be allocated in a 1:1 ratio through block randomization with random blocks of four, six, or eight. The randomization list was generated using R version 4.2.3 and the “blockrand” package [34]. The randomization list is overseen by an independent assessor.

#### Outcome measures

Relevant outcomes will be assessed at baseline, and all outcomes will be assessed 16-months post-intervention. To clarify, as two-month-old infants are breast/formula fed and non-ambulatory, diet, physical activity, and sleep are not assessed at baseline. Potential mediating effects and healthcare utilization will be assessed at baseline, mid-intervention (8 months), and post-intervention (16 months). At baseline the participating parent will also fill in a questionnaire including demographic questions for both parents.

##### Primary outcomes

Key dietary behaviours will be assessed using a modified version of the National Health and Welfare’s survey on health behaviours [35], as we have used previously [16]. The intake of fruits, vegetables, sweet treats, savoury treats, and sweetened beverages will be assessed by parents reporting the average number of standardized portions per day over the past two weeks. Children’s physical activity and sleep will be assessed using an ActiGraph wGT3x accelerometer (ActiGraph, Pensocola USA). Children will wear the accelerometer on their hip for seven consecutive days following a 24 h protocol. Parents will also be asked to fill in a sleep log with when their child goes to bed and wakes up (including naps). Diet, physical activity, and sleep will be assessed post-intervention. Screen time will be assessed at baseline, mid-intervention, and post-intervention where parents will report the average amount of screen time their infant/child has on weekdays and weekend days separately.

##### Secondary outcomes

Children’s weight (kg) and length (cm) at baseline (i.e., 2 months of age) and post-intervention (i.e., 18 months of age) will be reported by parents. Two and 18 months are standard visits at CHS, and parents will report their child’s weight and height at those visits including the date of the visit. Children’s BMI z-scores will be calculated using the World Health Organization’s growth standards [36]. Parental self-efficacy with regards to the parent’s own ability for promoting health behaviours will be assessed post-intervention using an electronic version of the `Parental self-efficacy for promoting healthy dietary and physical activity behaviours in their children´ questionnaire. The questionnaire includes 16 questions separated into three areas, self-efficacy for: (i) promoting healthy dietary behaviours; (ii) limit-setting of unhealthy behaviours; and (iii) promoting healthy physical activity behaviours in children [37].

##### Potential mediators

Targeted mediators of the intervention include parental knowledge related to diet, active play, and screen time and they will be assessed at baseline, mid-intervention (when the child is approximately 10 months old), and post-intervention through a digital questionnaire. This information will aid in elucidating potential pathways through which the intervention may have an effect.

#### Healthcare utilization

At baseline, mid-intervention, and post-intervention parents will be asked to report if they have had any extra visits to CHS or other healthcare services to discuss questions regarding their child’s weight, feeding, or movement behaviours. If they answered yes, they will be asked to further specify the number of visits, and who the visit(s) were with (e.g., CHS nurse, doctor, dietician) as well as if the visit was in person, digital, or through a telephone call.

#### Demographic information

Demographic information on both parents will be collected in the baseline questionnaire. This information includes age, sex, educational attainment, country of birth, height, and weight.

#### Sample size

The study was powered to detect a statistically significant effect of 9 gram in vegetable intake between the intervention group and control group, which corresponds to a small to moderate effect size. Vegetable intake was chosen as this is known to be the most difficult behaviour to change. A total of 352 study participants (176 in each group) is needed to detect a difference of 9 gram between the groups in a two-sided independent samples t-test with 80% statistical power at a significance level of 5% assuming an equal standard deviation of 32 g (corresponding to a Cohen’s d of 0.3) within each group, which comes from the 2021–2024 Swedish National Dietary Survey in 18 month year old children [4]. Accounting for a 30% expected drop-out rate, 504 individuals will be recruited for this study (252 per group). The sample size calculations were conducted using the statistical software R version 4.2.3 with the “pwr” package.

#### Data analysis

All analyses will follow intention-to-treat, i.e., including participants in the group they were randomly allocated to. Depending on observed outcome distributions, linear or generalized linear regression models will be fitted with primary and secondary outcomes as the dependent variable and the intervention group (intervention vs. control) as the independent variable.

All models will be adjusted for the child’s sex and age. The models with screen time and BMI z-scores as the dependent variable will also be adjusted for the respective baseline values. The estimated mean difference between groups with corresponding p-values (two-sided) and 95% confidence intervals will be presented. If there is a large proportion of children reporting zero values (e.g., for sweet and savoury treats, sweet drinks and screen time, which has been found previously [26]), additional analyses will be conducted to assess the effect of the Health4Life intervention on the odds of consuming these products or watching screens versus not consuming these products or watching screens.

#### Economic evaluation

A cost consequence analysis will be undertaken from a healthcare funder perspective to determine if the Health4Life intervention represents `value for money´ when measured incrementally against usual practice. Pathway analysis will identify all relevant intervention and control group activities, and costs, and resources will be measured using costs from trial records and published sources for the 2028 reference year. A `trial-based´ evaluation (i.e., costs/outcomes exactly per the trial) will be conducted using the primary outcomes. Sensitivity analyses will be undertaken, varying input parameters into the economic evaluation to gauge overall impact on the cost-effectiveness results. The economic evaluation will follow best-practice principles for conduct [38] and reporting [39]. A budget impact assessment will also be undertaken to inform the implementation and resource allocation decisions [40], informed by trial administrative data and the published literature.

### Implementation evaluation

The implementation of the Health4Life intervention will be explored in terms of its perceived acceptability, appropriateness, and feasibility among CHS nurses and parents in the intervention group.

#### Participants and data collection

##### Parents

Parental satisfaction with the Health4Life app will be assessed among those in the intervention group using a questionnaire modified from the MINISTOP 2.0 trial [16]. Frequencies and percentage will be used to assess satisfaction and the features/themes that were utilized. Furthermore, using purposive sampling parents in the intervention group will be asked if they are willing to participate in a semi-structured interview to evaluate their satisfaction and usage of the Health4Life app. For those that agree to participate an interview will be scheduled for a video/telephone interview at the parent’s convenience. The aim is to recruit 15 to 20 participants varying in ages, educational attainment, and country of birth. Objective usage data will also be collected to complement the self-reported and qualitative data. This will include how many times they have logged into the app as well as what features/content in the app they have used.

##### Child health service nurses

Semi-structured interviews will be conducted with CHS nurses to assess their perceived acceptability, appropriateness, and feasibility of implementing the Health4Life app in CHS. Using purposive sampling we will invite nurses participating in the Health4Life trial to participate in a semi-structured interview via email. The aim is to recruit nurses from different CHS centers and geographical locations.

### Ethics

This study is approved by the Swedish Ethical Review Authority (Dnr: 2024-07372-01; 2025-00542-02; 2025-01222-02) and follows the Declaration of Helsinki. Written informed consent is obtained from both parents for the randomized controlled trial and from participating parents and CHS nurses in the implementation evaluation.

### Trial status

The Health4Life trial was initiated in April 2025 and is progressing accordingly.

## Discussion

The Health4Life trial will evaluate whether a digital intervention (i.e., Health4Life app) for parents of young infants can help foster healthy diet and movement behaviours from the start of life. To date, there has been no trials starting in early infancy in Sweden; however, parent-focused interventions commencing in early infancy in other countries have been shown to be effective [12]. In Sweden, CHS reaches 99% of all children [19], and all CHS nurses maintain continuous contact with families through routine visits from birth until the age of five [30]. Furthermore, the Swedish National Board of Health and Welfare have identified that there is a need for more material, education, and working methods to promote healthy lifestyle behaviours within CHS [19]. They have also stated that the CHS system needs to develop and adjust their ways of working in line with societal development, as a way to meet current needs within public health. Parents of young children have expressed that they would like more support around diet and movement behaviours [17]; however, lack of time at routine visits has been found to be a barrier to the provision of more support to parents [18, 41]. Thus, this demonstrates the need for a scalable intervention to support parents with promoting health behaviours between visits at CHS, to complement the care they are already receiving.

To date, only one digital intervention has been evaluated to support parents in improving diet and physical activity behaviours in young children in Sweden. MINISTOP was developed with the overall aim to support parents in improving health behaviours in preschool aged children (from 2.5 to 4 years of age) in order to prevent overweight and obesity [15, 16]. In both the efficacy (MINISTOP 1.0) [15] and the effectiveness (MINISTOP 2.0) trials [16] statistically significant improvements in diet and screen time behaviours were observed in the intervention compared to the control group, with parents reporting high satisfaction and usage [41]. Furthermore, a type III implementation-effectiveness trial (MINISTOP 3.0) within CHS in Sweden has just been finalized in March 2026 [42]. Semi-structured interviews with parents and CHS nurses participating in MINISTOP 2.0 [41] and CHS nurses participating in MINISTOP 3.0 (unpublished data), have highlighted the need for a digital support tool to support healthy diet and movement behaviours starting already in infancy. Thus, should the Health4Life app prove to be effective, there is potential to integrate the two digital tools to provide parents with a complementary support system for health behaviours throughout the entire period during which their child attends CHS.

Strengths of this study include the type I hybrid design, which allows for the concurrent evaluation of effectiveness and implementation outcomes. Assessing these outcomes simultaneously has the potential to accelerate the translation of study findings into routine practice [20]. Traditionally, prevention research has followed a linear pathway from efficacy trials, progressing to effectiveness trials, and finally to broader dissemination [43]. However, in recent years there has been growing recognition of the need for greater flexibility in this research trajectory [44]. The Health4Life intervention is partly informed by the INFANT program developed in Australia, which has demonstrated effectiveness in improving health behaviours in early childhood [26]. In addition, MINISTOP has been shown to significantly improve health behaviours in both efficacy [15] and effectiveness [16] trials – an intervention similar to Health4Life but for children aged 2.5 to 4 years. This evidence, coupled with our qualitative findings indicating a need for Health4Life from both parents and CHS nurses [17, 18], motivate the use of an effectiveness-implementation design. This study is further strengthened through the randomized controlled study design (effectiveness component), planned large sample size, use of objective measures for physical activity and sleep, as well as the recruitment of families from CHS centres in four regions in Sweden (inclusive of both high and low socioeconomic areas), increasing generalizability. The use of co-design is also a notable strength of this study. To date, many public health interventions have used the top-down approach, and have not involved end-users in their development [45]. The Health4Life intervention has been designed in an iterative process in close collaboration with end-users (i.e., parents and CHS nurses) as well as other healthcare professionals (e.g., dieticians, dentists, and speech therapists). This is important as co-design has the potential to enhance the relevance, quality, and uptake of health interventions [46].

This study also has some limitations. Currently, the Health4Life app is only available in Swedish and English. This is a limitation, as approximately 27% of all children in Sweden have a migrant background [47], which could decrease the study’s generalizability. Furthermore, some of the outcomes are self-reported; however, this is very common in studies assessing health behaviours [48]. Nevertheless, it is important to highlight that when possible objective measures or validated self-reported measures are being used.

## Supplementary Information

Supplementary Material 1.

## Data Availability

No datasets were generated or analysed during the current study.

## Abbreviations

BMI: Body mass index
CHS: Child health service
mHealth: Mobile health
MINISTOP: Mobile-based intervention intended to stop obesity preschoolers
